# Ventricular fibrillation triggered by marijuana use in a patient with ischemic cardiomyopathy: a case report

**DOI:** 10.1186/1757-1626-1-373

**Published:** 2008-12-03

**Authors:** Adrian Baranchuk, Amer M Johri, Christopher S Simpson, Michelle Methot, Damian P Redfearn

**Affiliations:** 1 Arrhythmia Service, Kingston General Hospital, Queen's University Kingston, Ontario, Canada

## Abstract

**Background:**

A 60-year-old man presented to the emergency department with a left eye orbital rupture sustained during a fall due to syncope shortly after smoking more than his usual amount of marijuana.

**Case presentation:**

The patient reported experiencing a shock from his implantable cardioverter-defibrillator (ICD) device prior to the loss of consciousness. There was no biochemical, electrocardiographic, or clinical evidence of ischemia. ICD interrogation revealed one episode of ventricular fibrillation which was appropriately sensed and treated with a single shock of 35 Joules.

**Conclusion:**

Although the cardiovascular effects of marijuana are usually well tolerated in young healthy users, its use may trigger life-threatening arrhythmias in patients with structural heart disease. To the best of our knowledge, this is the first report on a case of ventricular fibrillation triggered by marijuana use in a patient with an ICD.

## Background

Marijuana (Cannabis) is one of the most widely used illicit drugs in North America [1]. Acute use is known to cause dose-dependent tachycardia, raised blood pressure, and increased cardiac output, however little is known about its effects on cardiac conduction [2-4]. Acute exposure has been linked to ventricular tachycardia (VT) in one previous report [5]. We report the first case of ventricular fibrillation (VF) occurring after more than usual exposure to marijuana recorded by an implantable cardioverter-defibrillator (ICD).

## Case report

A 60-year-old male presented to the hospital following a syncopal event that was preceded by an ICD shock shortly after exposure to marijuana.

The patient has a past history significant for a large anterior myocardial infarction and severe three-vessel coronary artery disease (CAD). The patient was not deemed a bypass candidate and has received a single chamber ICD (Medtronic EnTrust D154VRC) for prevention of sudden cardiac death (SCD) in context of a left ventricular ejection fraction of 20%.

The patient presented to hospital on this occasion following a left eye orbital rupture sustained during a fall due to the syncopal event shortly after smoking 4 "bongs" of marijuana. The patient experienced an ICD discharge prior to the loss of consciousness. There were no other associated symptoms. At no time did the patient have clinical signs or symptoms of angina or cardiac failure. The patient gives a history of experiencing an ICD discharge while smoking the same amount of marijuana 6 months prior. With both occasions, the patient had used 4 bongs rather than his usual 2 to 3 bongs of smoking. He did not seek medical attention at that time. Other than these two episodes of ICD discharge following more than his usual marijuana exposure, he denied any other syncope or ICD discharge.

There was no family history of SCD. The patient was receiving at home enteric coated aspirin 81 mg daily, carvedilol 25 mg twice daily, ramipril 10 mg once daily, atorvastatin 20 mg once daily, spironolactone 25 mg once daily, furosemide 40 mg once daily, diazepam 10 mg once daily, rabeprazole 10 mg as needed for heartburn, all without any changes in the two months prior to this episode. He denied any other recreational drug, or alcohol use. Home oxygen was used at night during sleep. The patient reports use of marijuana daily using a water pipe. He typically uses 7 or 8 "bongs" throughout the day, usually 2 to 3 "bongs" at one time. On the occasions that he inhaled a fourth "bong" consecutively, his defibrillator discharged.

**Figure 1:**
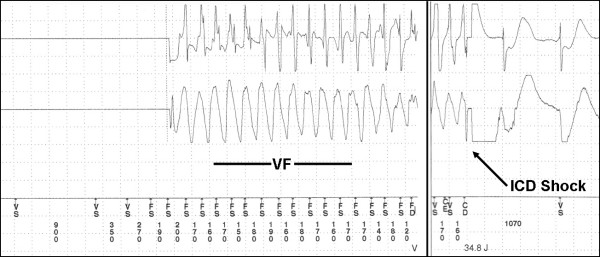
**Stored electrogram from the ICD.** Ventricular fibrillation followed by a successful ICD shock.

Investigations revealed no biochemical or electrocardiographic evidence of ischemia. Following repair of the globe rupture, the ICD was interrogated and revealed one event in the VF zone (rate > 188 bpm) which was adequately detected and treated with one appropriate shock. The device delivered anti-tachycardia pacing during charging and a 35 J shock (Figure 1) that was successful to restore normal sinus rhythm. During the interrogation, the device was reprogrammed to decrease the VT/VF detection from 12/16 to 6/8. The device was found to be otherwise functioning normally. The patient made an uneventful recovery and was discharged home after 2 days of intravenous amiodarone therapy and was discharged with a prescription for oral amiodarone and strong advice of quit smoking.

## Discussion

Although marijuana is the most frequently used illicit drug worldwide, its effects on the cardiovascular system remains controversial [6,7] (Table 1). In our case, the patient described experiencing 2 syncopal events, each occurring after more than his usual marijuana exposure. Although this observation does not prove a cause-and-effect relationship, the timing of the events suggests that dose dependent marijuana exposure may have lead to ventricular fibrillation in this patient with coronary artery disease and structural heart disease. We hypothesize that the arrhythmic event was triggered by excessive catecholamine release. Gash et al assessed the effects of smoking marijuana cigarettes containing 6 mg of delta-9-tetrahydrocannabinol in healthy men, using M-mode echocardiography. [3] The authors observed an increase in heart rate and left ventricular performance (mean rate of internal diameter shortening) for at least 1 hour after drug exposure compared to placebo cigarettes. After 39 minutes of marijuana exposure, plasma norepinephrine was significantly raised compared to control values and those after placebo cigarette use. Levels remained elevated for at least 2 hours after exposure, and suggested excessive sympathoadrenal discharge and prolonged catecholamine release was triggered by marijuana use. The authors concluded that marijuana had a positive inotropic effect on the heart in healthy subjects. In contrast, Prakash et al observed that marijuana use increased the heart rate and systolic and diastolic blood pressure and decreased the ejection fraction in men with CAD[4]. It has been suggested that in such individuals, augmentation of heart rate may lead to deterioration of left ventricular performance [3,5]. We therefore hypothesize that in this patient with significant heart disease, increased catecholamine release following increased marijuana exposure triggered ventricular fibrillation. The possibility remains that the arrhythmia could be unrelated to marijuana exposure however this seems unlikely as the events only occurred shortly after a similar amount of exposure on 2 separate occasions. The Naranjo score, a validated scoring tool to determine adverse drug reaction probability, revealed a probable relationship between marijuana and ventricular fibrillation (score of 5). [8]

**Table 1:** Marijuana cardiovascular effects.

**System affected**	**Clinical implications**	**Comments**
Autonomic Nervous System: Increase norepinephrine release	Sinus tachycardia Hypertension	Healthy volunteers

Increased sinus automaticity	Sinus tachycardia	Healthy volunteers

Facilitated conduction velocity	Supraventricular and/or ventricular tachycardia, PVC	Healthy volunteers/structural heart disease

Vascular periphery: dysfunctional baroreceptor activity	Vasovagal syncope/hypotension	

Coronary arteries	Coronary artery disease (AMI)	Controversial

Central Nervous system	Stroke	Controversial

**PVC: **premature ventricular contraction; **AMI: **acute myocardial infarction

An alternative mechanism for arrhythmia has been postulated in one previous report describing a case of ventricular tachycardia following habitual marijuana use. A healthy 34 year old man presented with VT three hours after smoking marijuana [5]. The patient underwent successful electrical cardioversion. Coronary angiography revealed normal epicardial vessels without stenosis; however a marked reduction in coronary flow was detected. The clinical tachycardia was also inducible in the electrophysiology laboratory. Following verapamil therapy, his coronary flow normalized and VT was no longer inducible. In this patient the VT had a right bundle-branch block and left axis deviation morphology (Belhassen morphology). The VT responded well to verapamil therapy. Marijuana may have played a role by enhancing triggered activity in the Purkinje fibers. In our case, the clinical manifestation was VF; thereby the physiopathology mechanism described for VT is less suitable.

## Conclusion

It is strongly suspected that increased use of marijuana in our patient with structural heart disease, lead to this syncopal event and ventricular fibrillation as recorded by the patient's ICD. Chronic users of marijuana with known structural heart disease should be cautioned on increasing their habitual marijuana use.

## Consent

Written informed consent was obtained from the patient for publication of this case report and accompanying images. A copy of the written consent is available for review by the Editor-in-Chief of this journal.

## Competing interests

The authors declare that they have no competing interests.

## Authors' contributions

All authors have contributed to preparation of the manuscript. AB and AJ have written the manuscript. MM has supervised the aspects related to pharmacodynamics of Marijuana. CS have supervised the ICD functioning. DR has critically reviewed the manuscript and helped in the preparation of the figures.
